# Supplementary figures and images for: A novel chaperone-effector-immunity system identified in uropathogenic Escherichia coli UMN026
Source: PeerJ. 2024 May 20;12:e17336. doi: 10.7717/peerj.17336 (PMC11114119; doi:10.7717/peerj.17336)

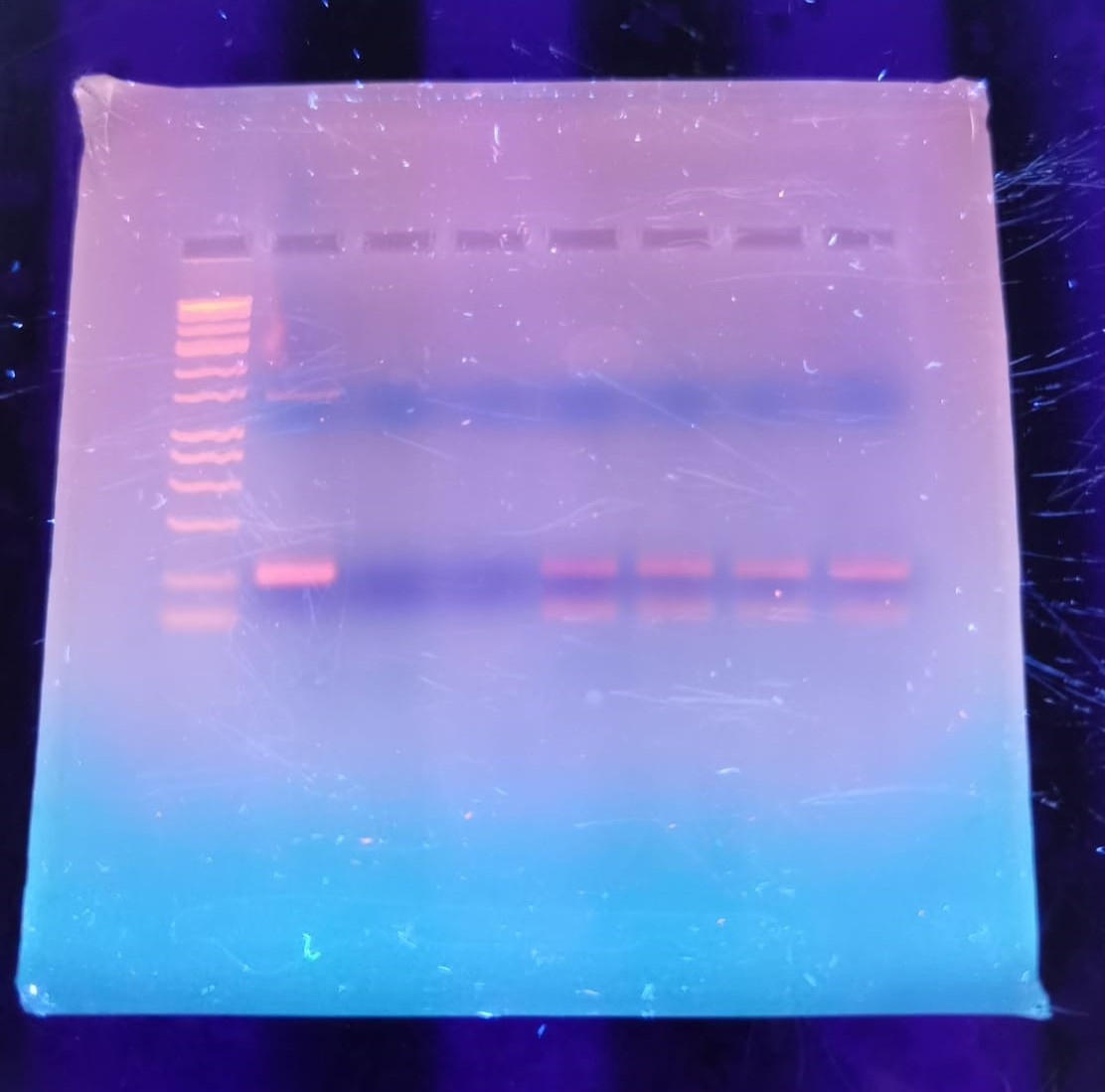

Supplement: Supplemental Information 2 [file peerj-12-17336-s002.zip › Fig 2A.jpeg]

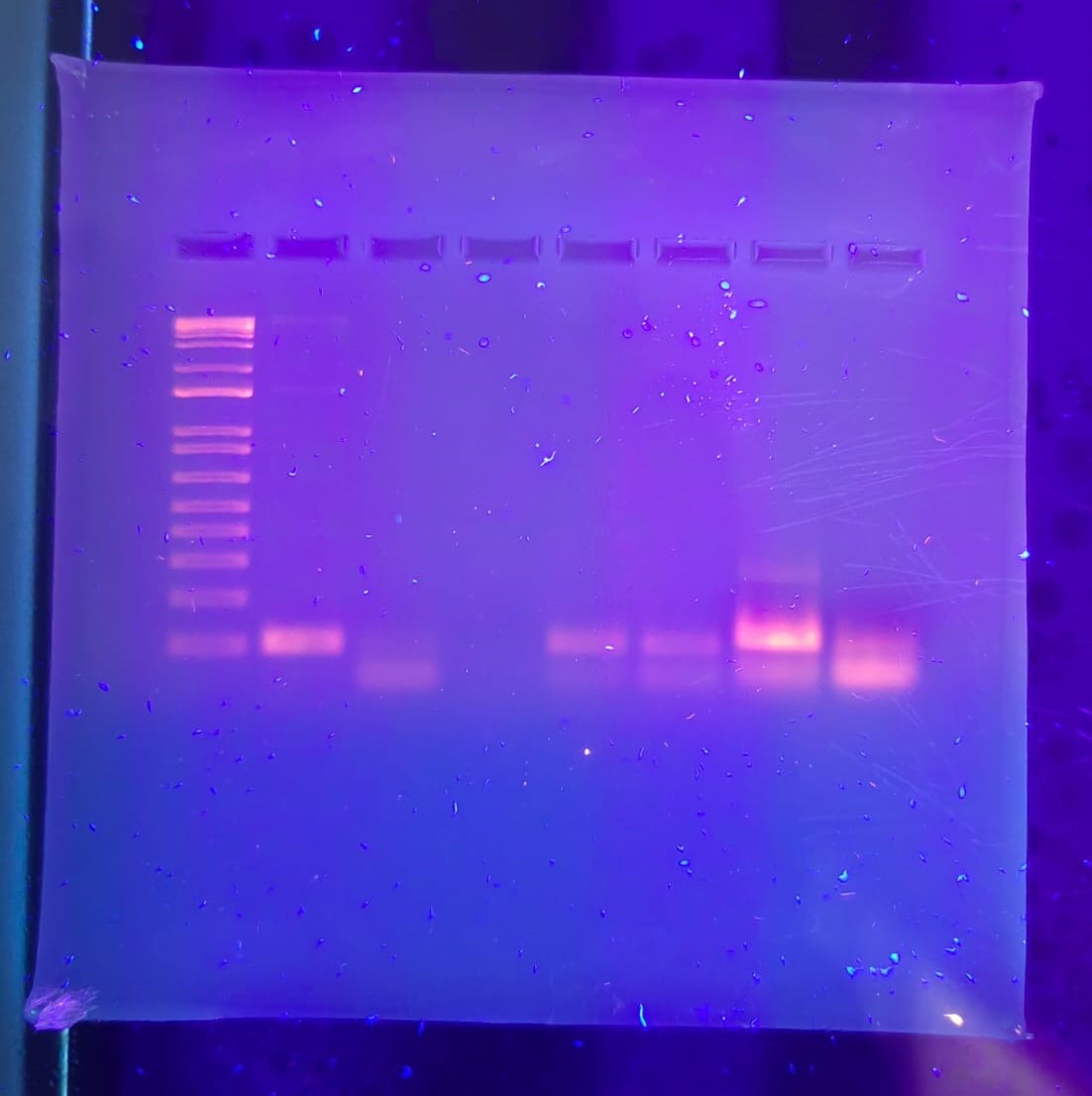

Supplement: Supplemental Information 2 [file peerj-12-17336-s002.zip › Fig 2B.jpeg]

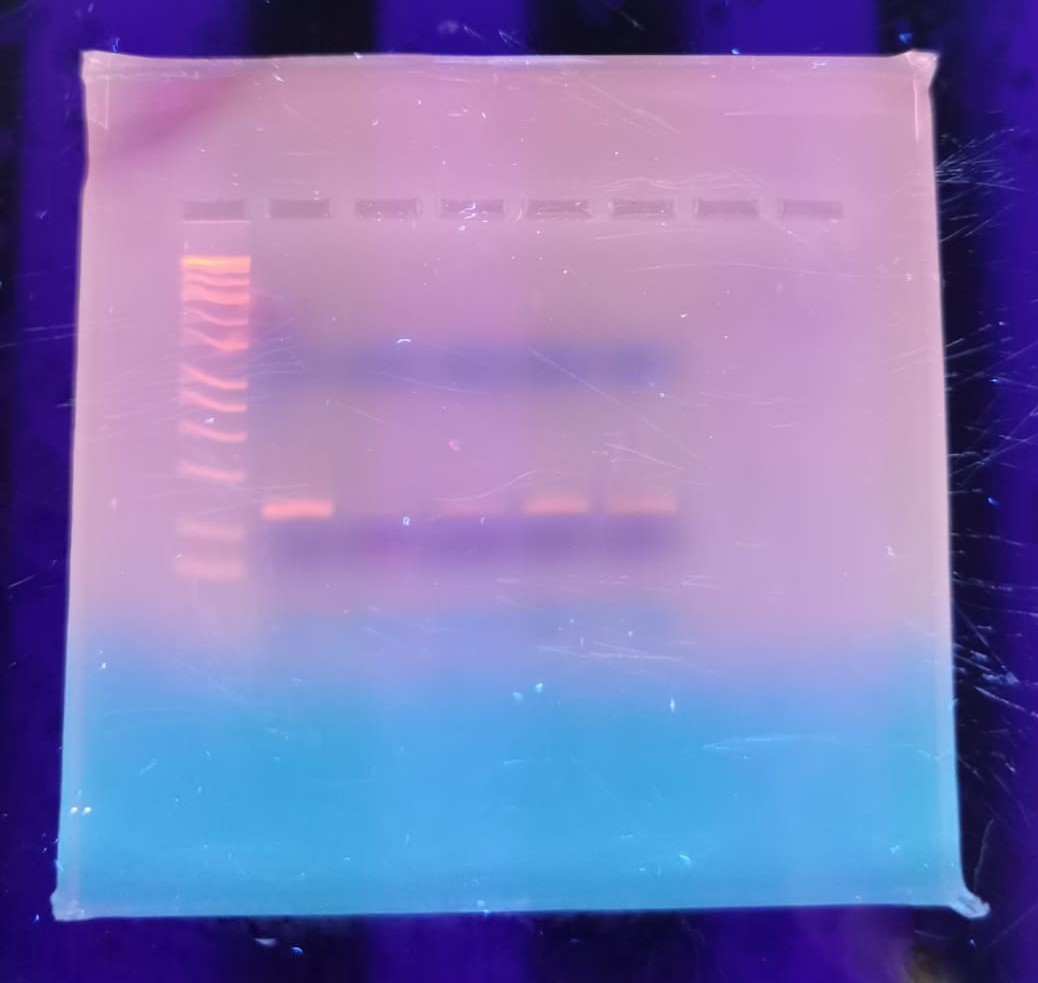

Supplement: Supplemental Information 2 [file peerj-12-17336-s002.zip › Fig 2C.jpeg]

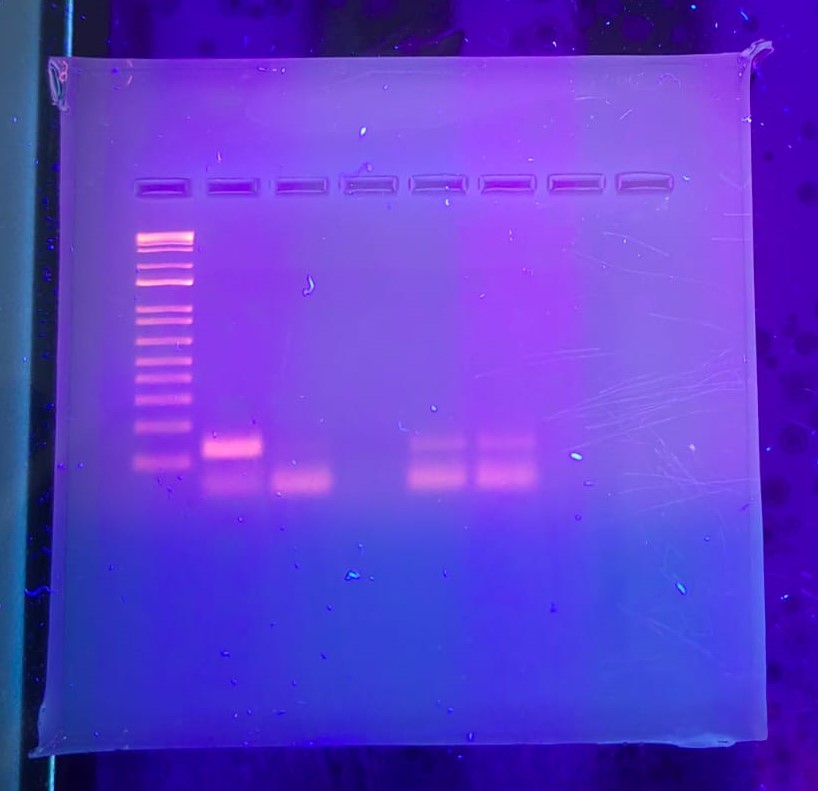

Supplement: Supplemental Information 2 [file peerj-12-17336-s002.zip › Fig 2D.jpeg]

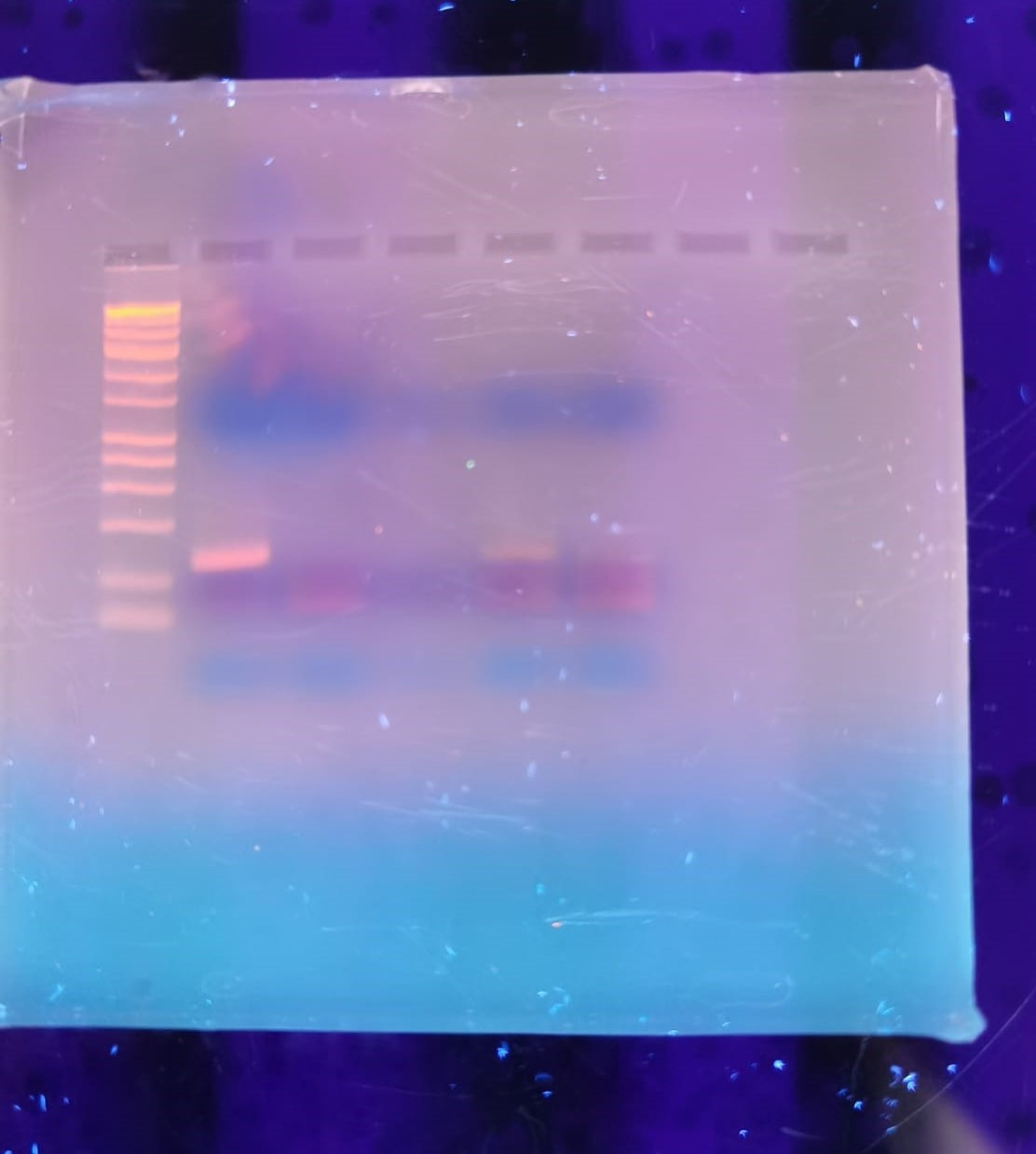

Supplement: Supplemental Information 2 [file peerj-12-17336-s002.zip › Fig 2E.jpeg]

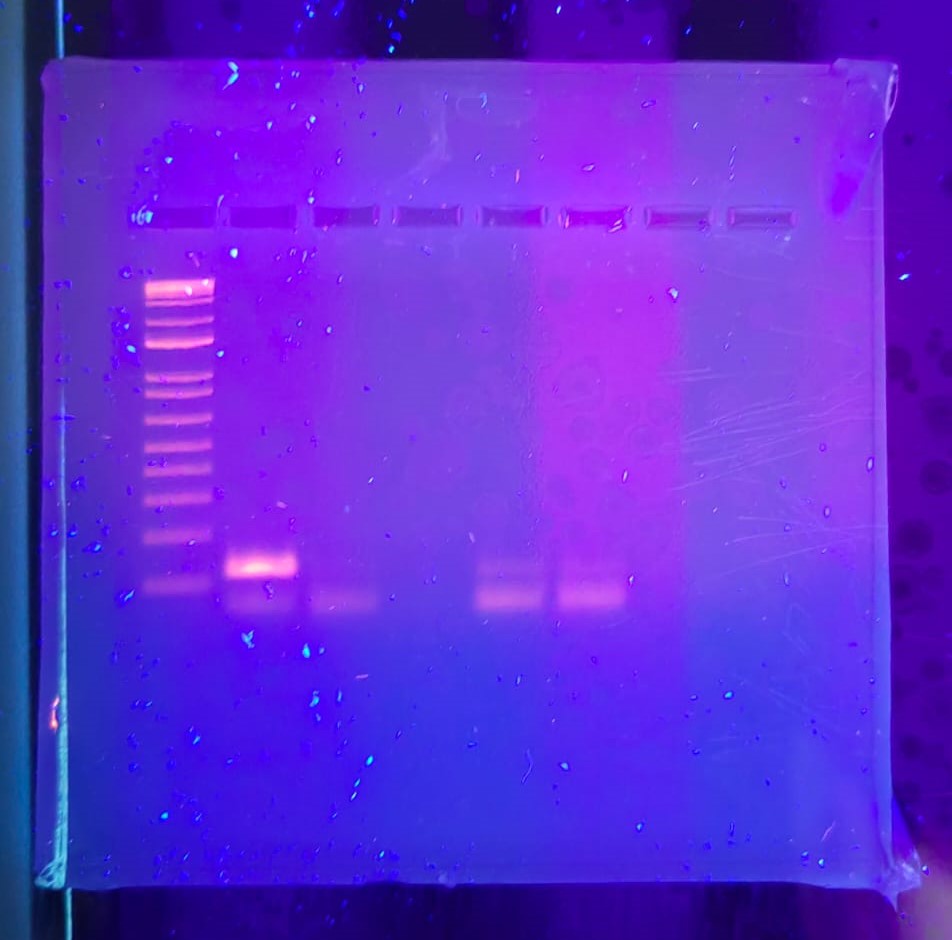

Supplement: Supplemental Information 2 [file peerj-12-17336-s002.zip › Fig 2F.jpeg]

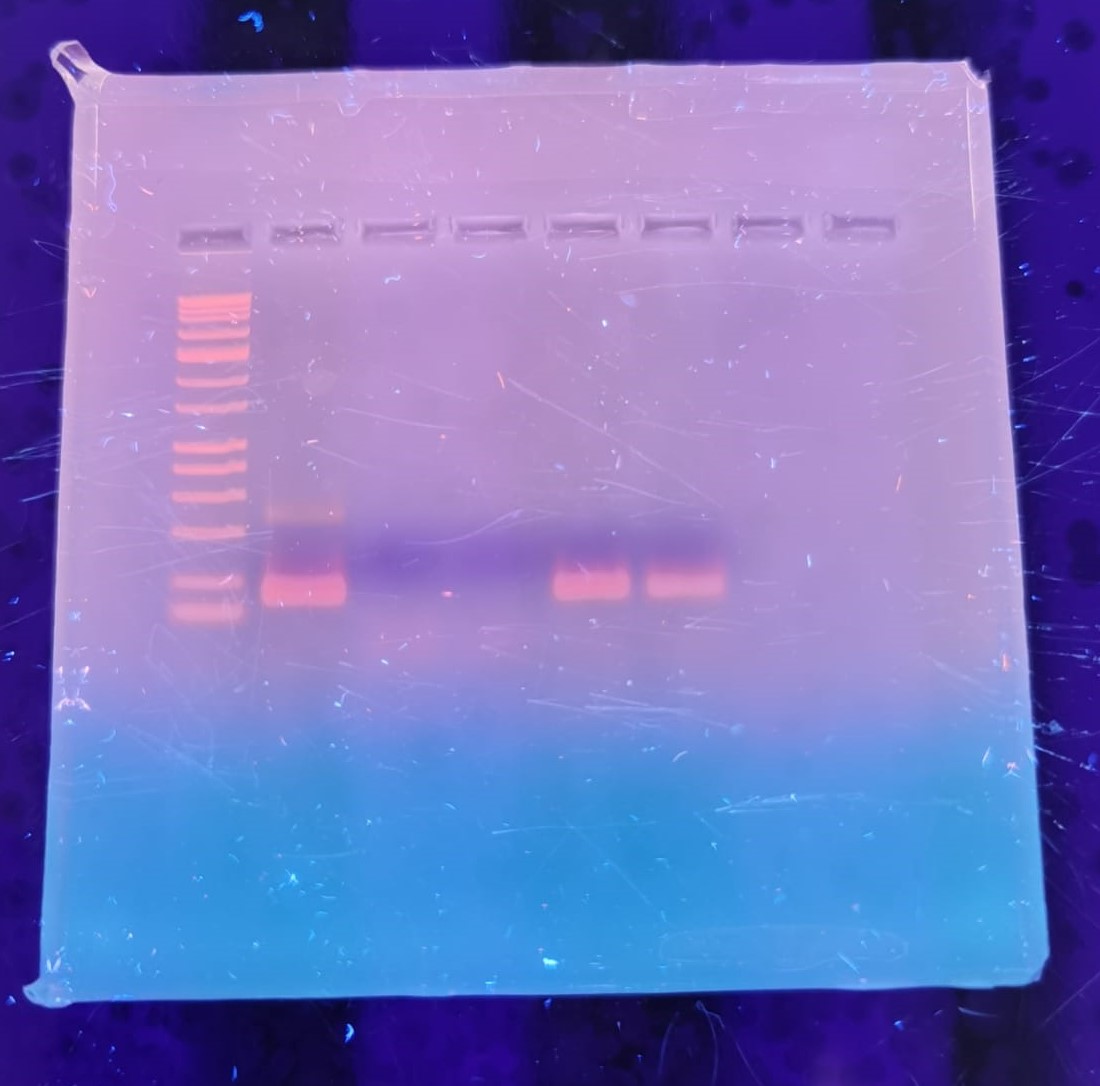

Supplement: Supplemental Information 2 [file peerj-12-17336-s002.zip › Fig 2G.jpeg]

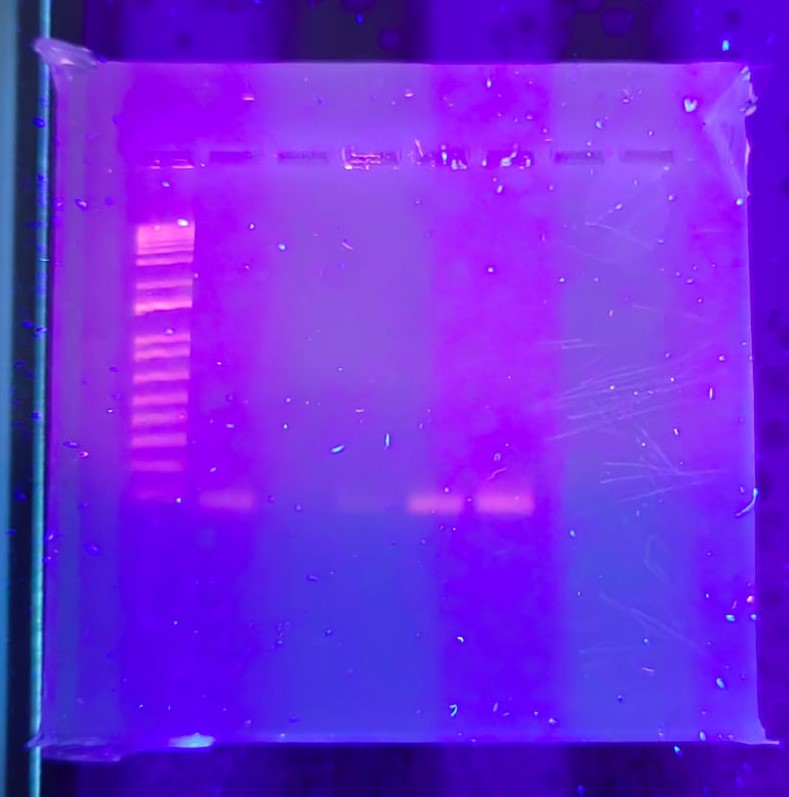

Supplement: Supplemental Information 2 [file peerj-12-17336-s002.zip › Fig 2H.jpeg]

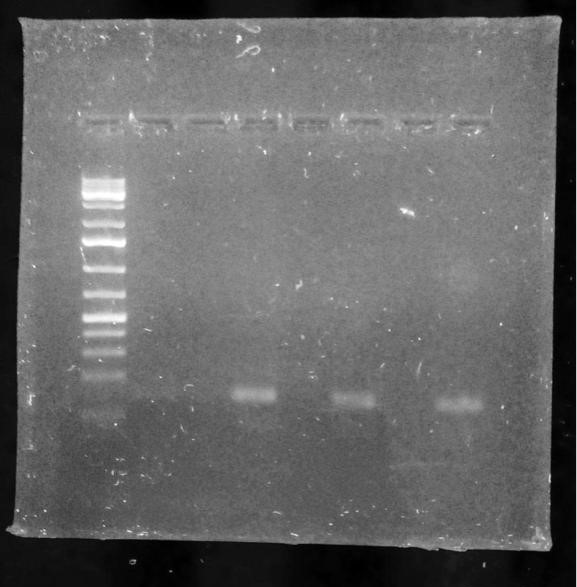

Supplement: Supplemental Information 2 [file peerj-12-17336-s002.zip › Fig 3A.jpeg]

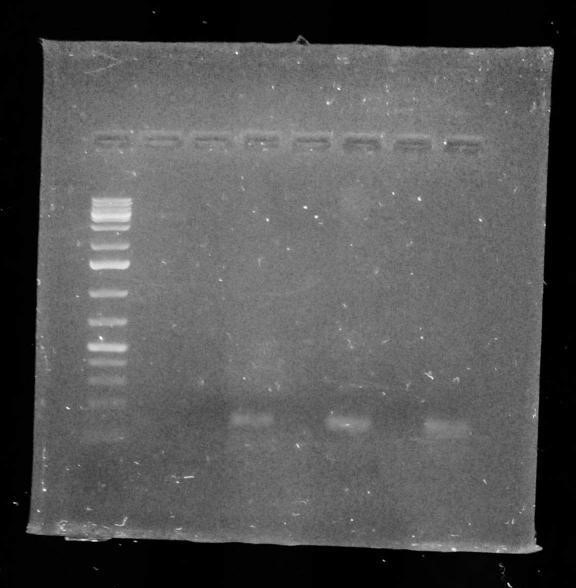

Supplement: Supplemental Information 2 [file peerj-12-17336-s002.zip › Fig 3B.jpeg]

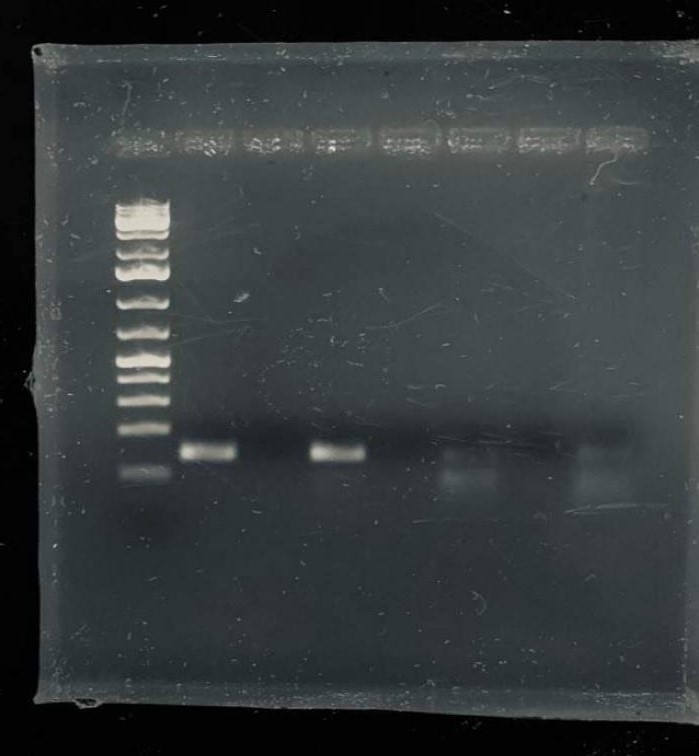

Supplement: Supplemental Information 2 [file peerj-12-17336-s002.zip › Fig 3C.jpeg]

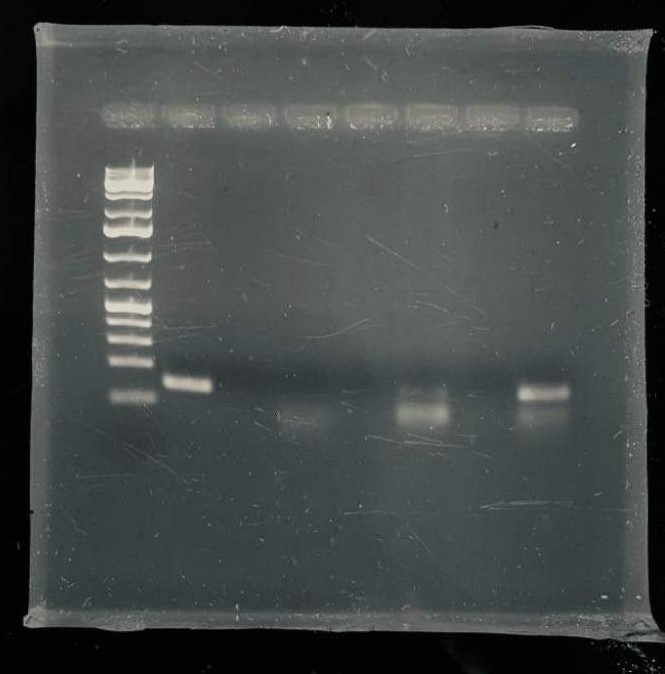

Supplement: Supplemental Information 2 [file peerj-12-17336-s002.zip › Fig 3D.jpeg]

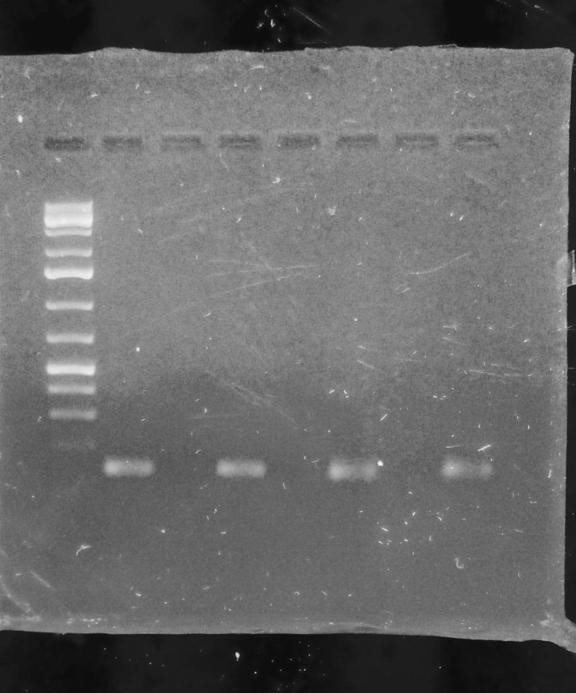

Supplement: Supplemental Information 2 [file peerj-12-17336-s002.zip › Fig 4A.jpeg]

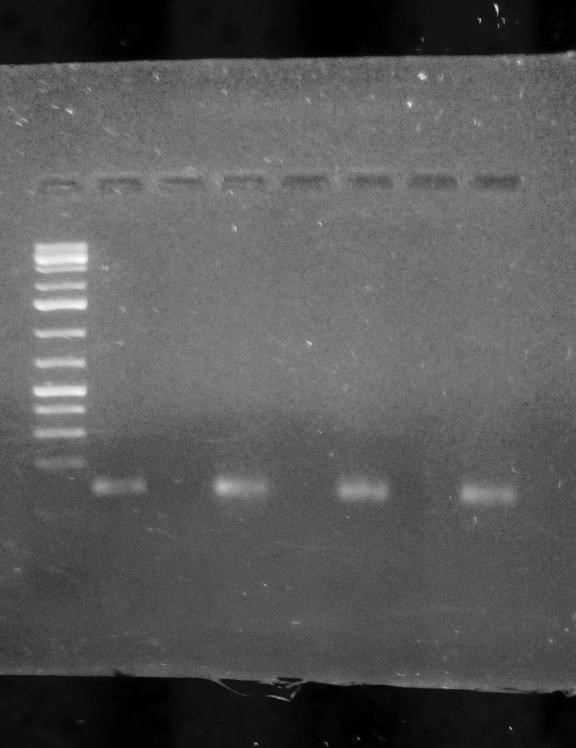

Supplement: Supplemental Information 2 [file peerj-12-17336-s002.zip › Fig 4B.jpeg]

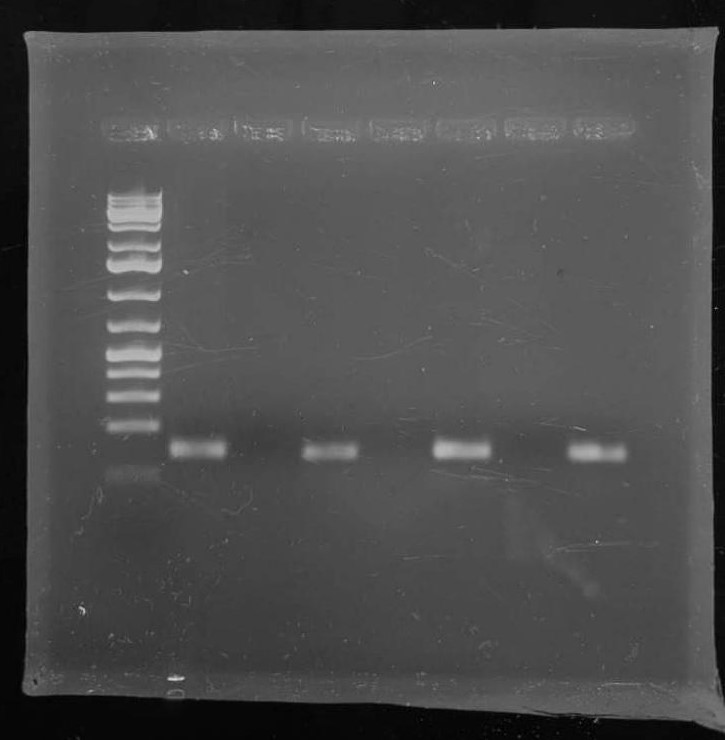

Supplement: Supplemental Information 2 [file peerj-12-17336-s002.zip › Fig 4C.jpeg]

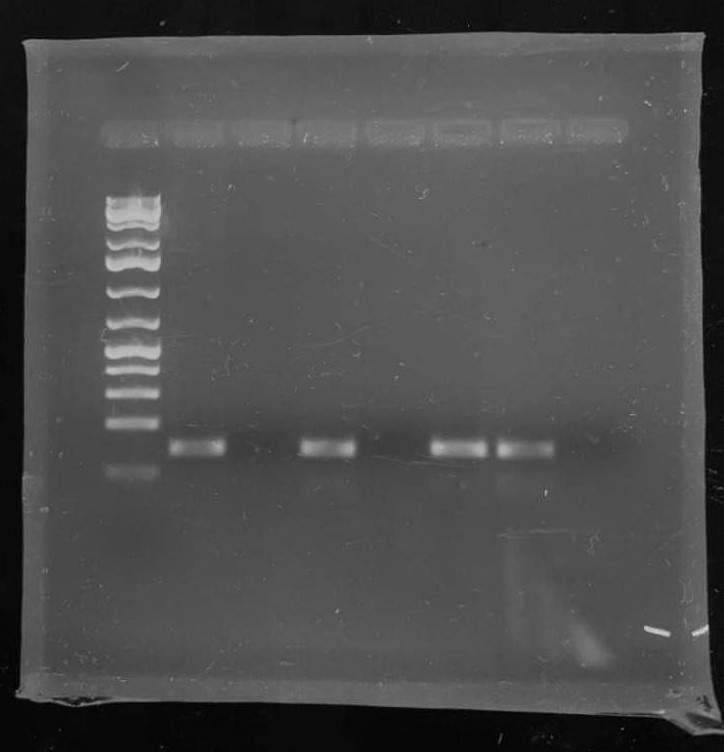

Supplement: Supplemental Information 2 [file peerj-12-17336-s002.zip › Fig 4D.jpeg]

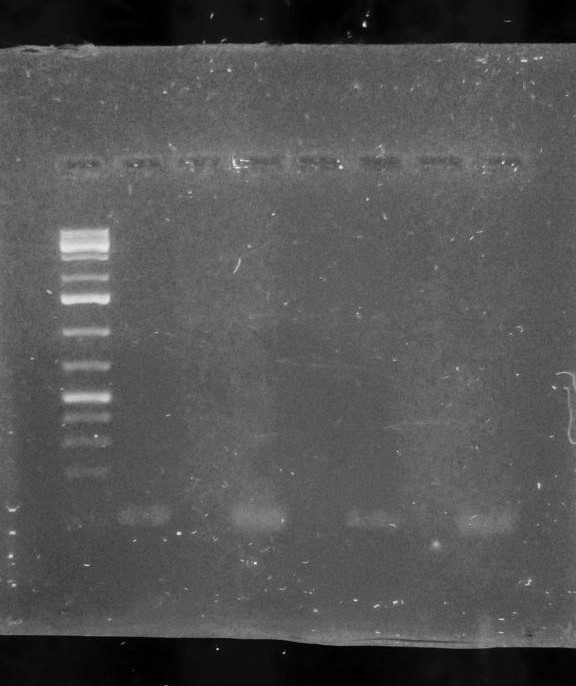

Supplement: Supplemental Information 2 [file peerj-12-17336-s002.zip › Fig 5A.jpeg]

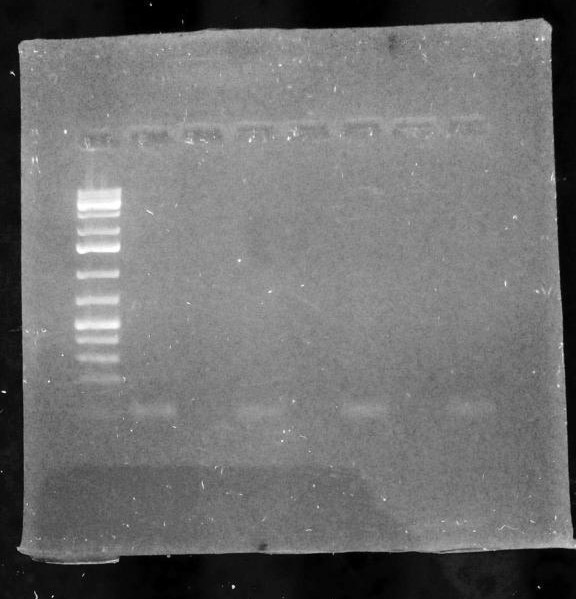

Supplement: Supplemental Information 2 [file peerj-12-17336-s002.zip › Fig 5B.jpeg]

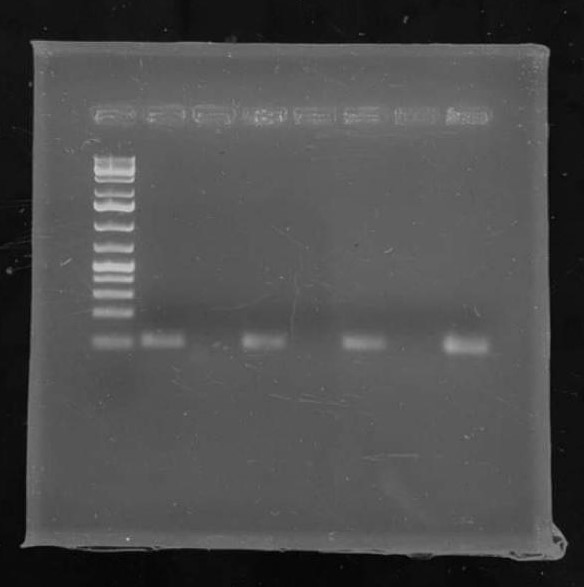

Supplement: Supplemental Information 2 [file peerj-12-17336-s002.zip › Fig 5C.jpeg]

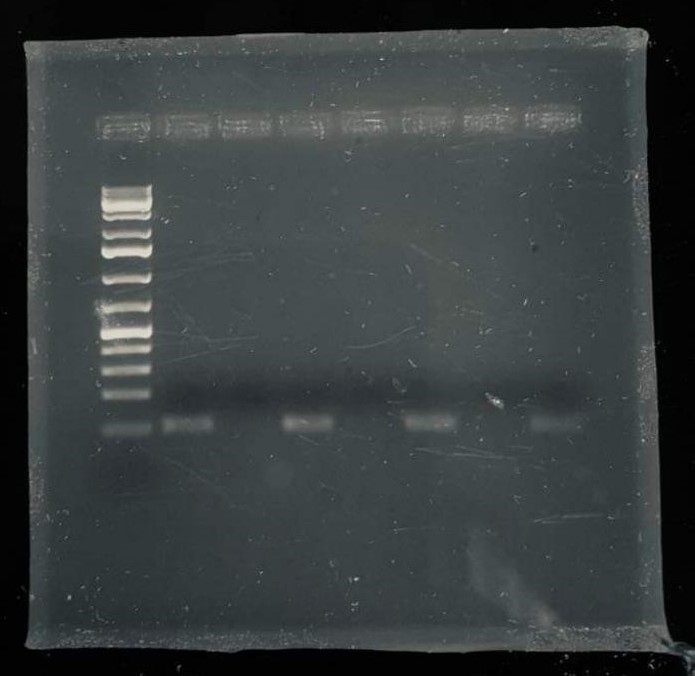

Supplement: Supplemental Information 2 [file peerj-12-17336-s002.zip › Fig 5D.jpeg]

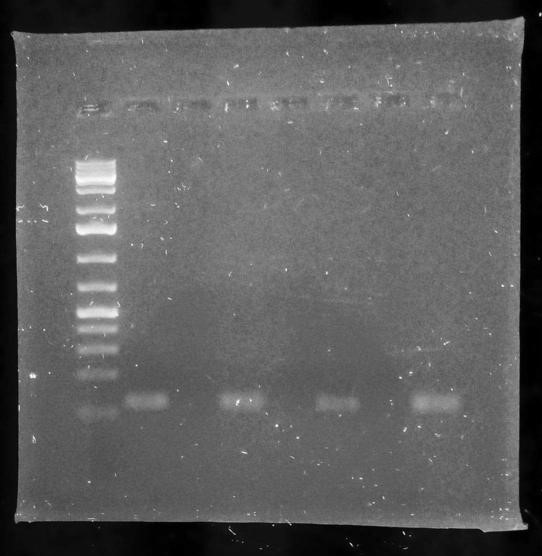

Supplement: Supplemental Information 2 [file peerj-12-17336-s002.zip › Fig 6A.jpeg]

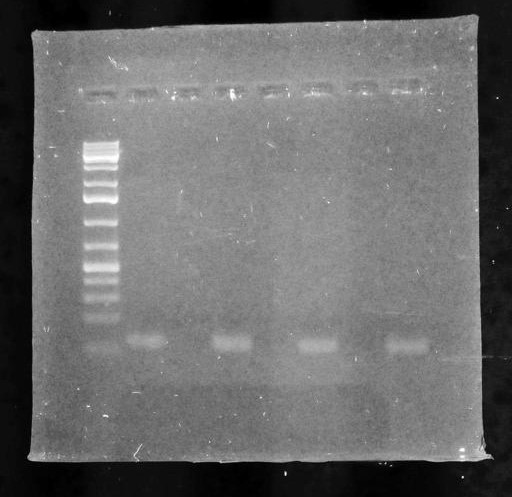

Supplement: Supplemental Information 2 [file peerj-12-17336-s002.zip › Fig 6B.jpeg]

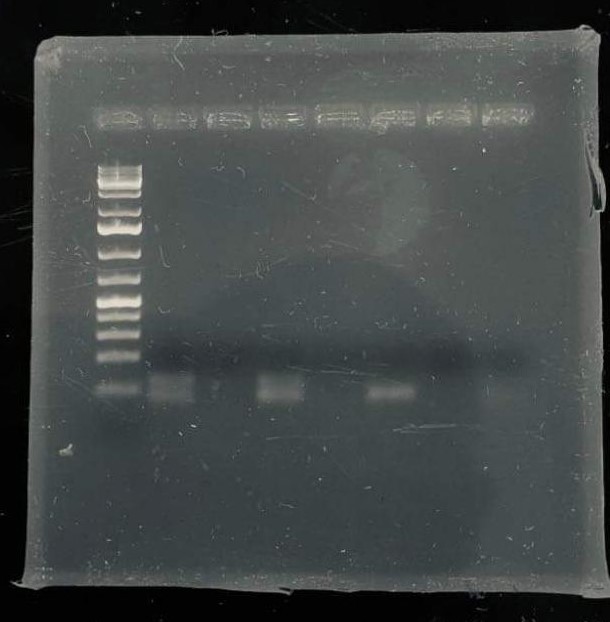

Supplement: Supplemental Information 2 [file peerj-12-17336-s002.zip › Fig 6C.jpeg]

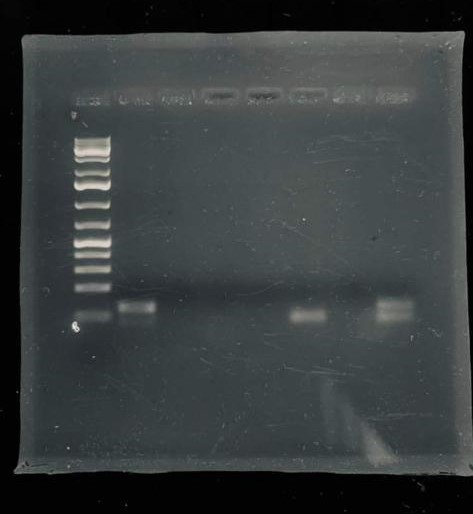

Supplement: Supplemental Information 2 [file peerj-12-17336-s002.zip › Fig 6D.jpeg]

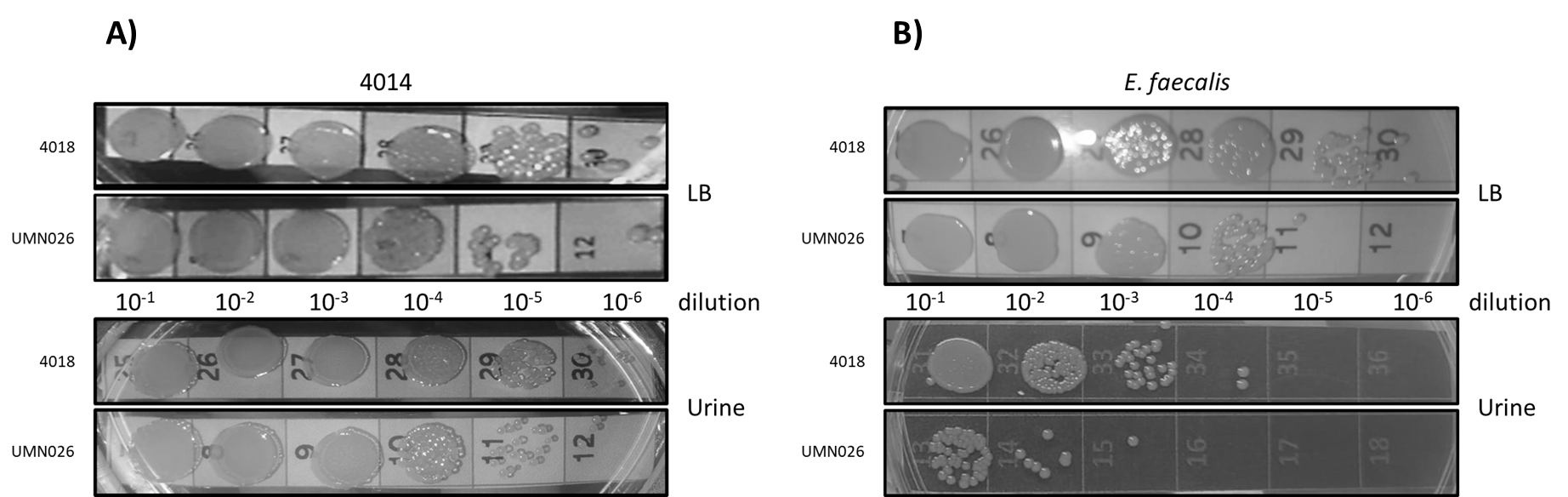

Supplement: Supplemental Information 4 — The bacterial growth at different dilutions (10-1 - 10-6) of the prey strains (A) UPEC 4014 and (B) E. faecalis after the interaction with attacking strains UPEC UMN026 and the negative control UPEC 4018 in LB and urine. [file peerj-12-17336-s004.png]
